# Axon guidance molecules in immunometabolic diseases

**DOI:** 10.1186/s41232-021-00189-0

**Published:** 2022-01-19

**Authors:** Yoshimitsu Nakanishi, Sujin Kang, Atsushi Kumanogoh

**Affiliations:** 1 Department of Respiratory Medicine and Clinical Immunology, Graduate School of Medicine, Osaka University, Suita City, Osaka, 565-0871 Japan; 2 Department of Immunopathology, Immunology Frontier Research Center, Osaka University, Suita City, Osaka, 565-0871 Japan; 3 Integrated Frontier Research for Medical Science Division, Institute for Open and Transdisciplinary Research Initiatives (OTRI), Osaka University, Suita City, Osaka, 565-0871 Japan; 4 Department of Advanced Clinical and Translational Immunology, Graduate School of Medicine, Osaka University, Suita City, Osaka, 565-0871 Japan; 5 Department of Immune Regulation, Immunology Frontier Research Center, Osaka University, Suita City, Osaka, 565-0871 Japan; 6 Center for Infectious Diseases for Education and Research (CiDER), Osaka University, Suita, Osaka, 565-0871 Japan

**Keywords:** Semaphorins, Plexins, Neuropilins, Chronic inflammation, Obesity, Diabetes, Atherosclerosis

## Abstract

The global prevalence of metabolic diseases, such as obesity, diabetes, and atherosclerosis, is rapidly increasing and has now reached epidemic proportions. Chronic tissue inflammation is a characteristic of these metabolic diseases, indicating that immune responses are closely involved in the pathogenesis of metabolic disorders. However, the regulatory mechanisms underlying immunometabolic crosstalk in these diseases are not completely understood. Recent studies have revealed the multifaceted functions of semaphorins, originally identified as axon guidance molecules, in regulating tissue inflammation and metabolic disorders, thereby highlighting the functional coupling between semaphorin signaling and immunometabolism. In this review, we explore how semaphorin signaling transcends beyond merely guiding axons to controlling immune responses and metabolic diseases.

## Background

The prevalence of metabolic diseases, such as obesity, diabetes, and atherosclerosis, is rapidly increasing, despite extensive research into their pathogenesis. Although chronic tissue inflammation indicates the involvement of immune responses in the pathogenesis of metabolic disorders, the regulatory mechanisms of immunometabolic crosstalk in these diseases are not fully understood.

Semaphorins are a large family of secreted and membrane-associated molecules that are essential for the development of the nervous system. The semaphorin family, which is characterized by an extracellular N-terminal Sema domain and plexin–semaphorin–integrin domains, is composed of eight subclasses [1]. Classes I–II and VIII represent invertebrate and virus-encoded semaphorins, respectively. Vertebrate semaphorins are grouped into classes III–VII. Semaphorins exert pathophysiological functions mainly through plexins and neuropilins (Nrps). Accumulating evidence has indicated that semaphorin signaling plays a pivotal role in various pathophysiological processes outside the nervous system [2, 3]. Recent studies have focused on the functions of semaphorin signaling in immunometabolic regulation and disorders, such as obesity, diabetes, and atherosclerosis [4–6]. In addition, we recently found that Sema6D signaling induces lipid metabolic reprogramming that is indispensable for anti-inflammatory polarization of macrophages, thus highlighting an essential link between semaphorin signaling and lipid metabolism [7]. This review summarizes recent advances in understanding metabolic diseases, with particular focus on the role of semaphorin signaling in the development of obesity, diabetic complications, and atherosclerosis.

### Role of semaphorins in adipose tissue inflammation

**Fig. 1:**
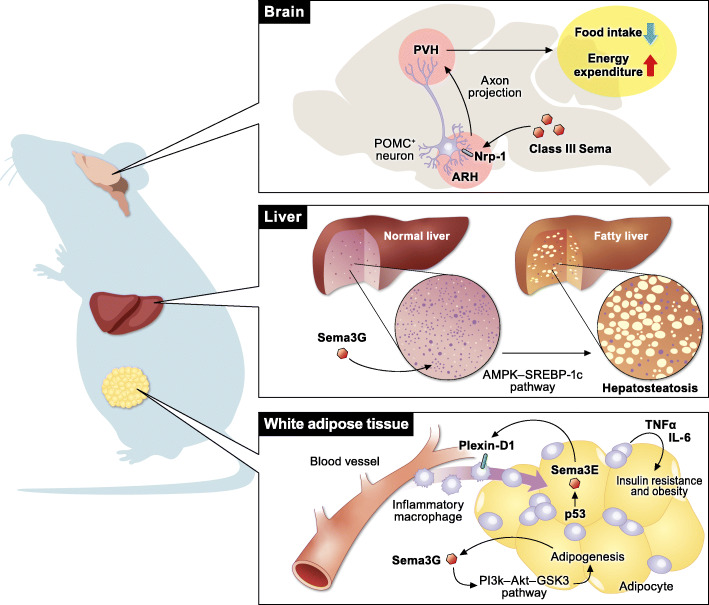
Multifaceted functions of class III semaphorins in the regulation of systemic metabolism. Sema3E recruits inflammatory macrophages into white adipose tissues via Plexin-D1 and promotes obesity. Sema3G promotes adipogenesis not only in the adipose tissue but also in the liver. In addition to metabolic regulation in the peripheral tissues, class III semaphorins regulate the development of hypothalamic melanocortin circuits, which are essential for systemic energy homeostasis

Among the semaphorin subclasses, class III semaphorins are the most widely studied molecules in the regulation of obesity and systemic metabolism (Fig. 1). In 2013, Shimizu et al. [8] revealed the pivotal role of Sema3E in regulating adipose tissue inflammation and systemic insulin resistance. In mice, a high-fat/high-sucrose diet enhances Sema3E and Plexin-D1 expression in adipocytes and macrophages, respectively. In addition, the plasma level of Sema3E is elevated in patients with diabetes. The Sema3E–Plexin-D1 signaling axis promotes inflammatory macrophage infiltration into visceral white adipose tissues, leading to adipose tissue inflammation and insulin resistance. The soluble form of Plexin-D1, which binds to Sema3E and inhibits its activity, markedly suppresses inflammation in adipose tissues and improves insulin resistance. In contrast, the overexpression of Sema3E in adipose tissues exacerbates adipose tissue inflammation and insulin resistance through enhanced accumulation of inflammatory macrophages in visceral white adipose tissues. Based on these findings, Yoshida et al. [9] developed a peptide vaccine targeting Sema3E as a therapeutic tool for diet-induced obesity. In this study, a peptide corresponding to amino acids 359–368 (HKEGPEYHWS) of Sema3E was conjugated to keyhole limpet hemocyanin and was used as a peptide vaccine for Sema3E. In mice, administration of this peptide vaccine induced Sema3E antibody production and suppressed the infiltration of Plexin-D1 expressing cells, leading to the amelioration of visceral adipose tissue inflammation and systemic glucose intolerance. Overall, the Sema3E–Plexin-D1 signaling axis is a promising therapeutic target for diet-induced obesity.

Sema3G, another class III semaphorin, plays key roles in adipogenesis [10]. Sema3G expression was markedly increased during adipocyte differentiation. Exogenous expression of Sema3G promotes adipogenesis, whereas Sema3G knockdown by shRNA suppresses adipocyte differentiation. Neutralizing antibodies against Nrp-2 block the effect of Sema3G on adipogenesis, suggesting that the Sema3G–Nrp-2 signaling axis is essential for adipogenesis in vitro. In mice, Sema3G activates the phosphatidylinositol-3 kinase (PI3K)–Akt–glycogen synthase kinase 3 (GSK3) and AMP-activated protein kinase (AMPK)–sterol regulatory element binding protein-1c (SREBP-1c) signaling pathways in adipose tissues and the liver, respectively, leading to obesity and insulin resistance. Therefore, knockdown of Sema3G by shRNA ameliorates obesity, hepatosteatosis, and insulin resistance. Moreover, serum levels of Sema3G are elevated in individuals with obesity [10].

In addition to their functions in adipose tissues, class III semaphorins regulate systemic energy balance by modulating hypothalamic neural circuits. Klaauw et al. [11] reported 40 rare heterozygous variants of genes encoding class III semaphorins as well as their receptors and co-receptors that are enriched in individuals with severe early-onset obesity. These rare variants disrupt the bioactivity of class III semaphorins, suggesting the essential role of class III semaphorin signaling in regulating systemic metabolism. In zebrafish, the deletion of seven class III semaphorin-coding genes increases somatic growth and adiposity. Class III semaphorin–Nrp signaling directs the development of pro-opiomelanocortin (POMC) axonal projections from the arcuate nucleus of the hypothalamus (ARH) to the paraventricular nucleus of the hypothalamus (PVH), which regulates feeding and energy expenditure. Thus, mice that lack Nrp-2 in POMC-expressing neurons exhibit obesity and reduced energy expenditure due to disrupted arcuate POMC axonal projections to the PVH.

With respect to the other semaphorin classes, the Sema6A–Plexin-A4 signaling axis regulates homeostatic energy expenditure and thermogenesis of brown adipose tissue (BAT) by modulating sympathetic innervation. Wolf et al. [12] revealed that methyl-CpG-binding protein 2 (MeCP2), a nuclear transcriptional regulator, modulates interactions between macrophages and neurons via the suppression of Sema6A–Plexin-A4 signaling, and thereby regulates metabolic homeostasis of BAT. In mice, macrophage-specific *Mecp2* deficiency results in spontaneous obesity due to decreased sympathetic innervation in BAT. *Mecp2*^*−/−*^ macrophages overexpress Plexin-A4, which suppresses the axonal outgrowth of Sema6A-expressing sympathetic nerves and innervation in BAT. Although the mechanism by which MeCP2 suppresses Plexin-A4 expression in macrophages remains unclear, this study revealed the essential roles of interactions between macrophages and neurons via the Sema6A–Plexin-A4 signaling axis in the regulation of metabolic homeostasis.

### Role of semaphorins in diabetic complications

**Fig. 2:**
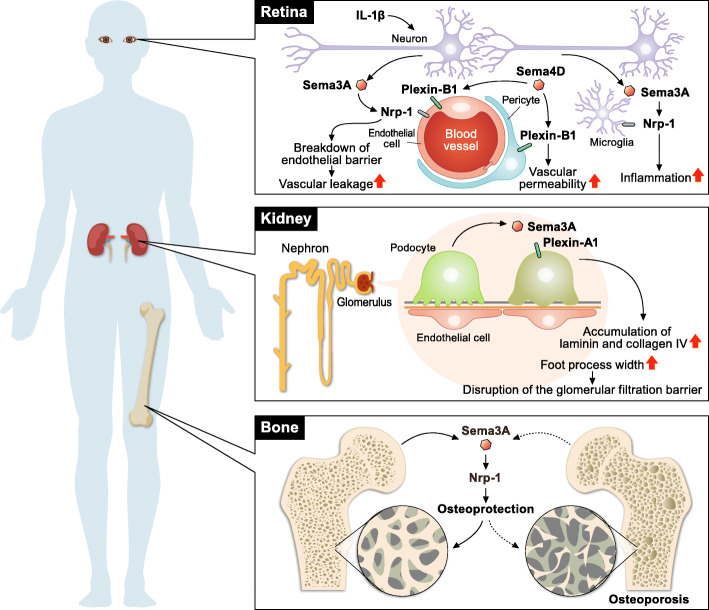
Role of semaphorins in the development of diabetic complications. Sema3A exaggerates diabetic retinopathy via the breakdown of retinal endothelial cell barrier function and recruitment of proangiogenic microglia. In the kidney, Sema3A disrupts the glomerular filtration barrier, contributing to the development of diabetic nephropathy. In contrast, Sema3A exerts osteoprotective effects, leading to the suppression of diabetic osteoporosis

Accumulating evidence has elucidated the mechanisms underlying the pathogenesis of diabetes, leading to the clinical application of various drugs, such as metformin, sulfonylureas, glinides, thiazolidinediones, dipeptidyl peptidase-4 inhibitors, glucagon-like peptide-1 receptor agonists, sodium-dependent glucose cotransporter 2 inhibitors, and insulin [13]. However, it remains challenging to understand the pathogenesis of diabetic complications, including neuropathy, retinopathy, and nephropathy. In this section, we provide an overview of the mechanism by which semaphorin signaling regulates diabetic complications (Fig. 2).

Diabetic retinopathy (DR) is a common complication associated with diabetic macular edema, vitreous hemorrhage, traction retinal detachment, and macular ischemia [14]. Although DR progression leads to vision loss, only limited effective treatments are currently available. The Sema3A–Nrp-1 signaling axis contributes to the development of diabetic retinopathy via multiple mechanisms. The levels of SEMA3A are elevated in the vitreous of patients with DR [15, 16]. In mice, streptozotocin-induced diabetes or oxygen-induced retinopathy upregulates Sema3A expression in the ganglion cell layer [15–17]. Neuron-derived Sema3A induces vascular leakage via the breakdown of retinal endothelial cell barrier function [15]. IL-1β induces Sema3A expression in the ischemic avascular retina [17]. The binding of Sema3A to Nrp-1 activates Src kinase and FAK in retinal microvascular endothelial cells, leading to the disruption of endothelial tight junctions and breakdown of the blood–retinal barrier [15]. Sema3A produced by hypoxic retinal ganglion cells also repels neo-vessels toward the vitreous [17]. In addition to the regulation of retinal vascular functions, Sema3A acts as a chemoattractant for proangiogenic microglia via Nrp-1, leading to vascular degeneration and pathological pre-retinal neovascularization [16]. Moreover, intravitreal administration of soluble Nrp-1, which neutralizes Sema3A bioactivity, reduces microglial infiltration and pathological neovascularization in retinopathy. Collectively, these studies indicate that the Sema3A–Nrp-1 signaling axis is a promising therapeutic target for retinopathy.

In contrast, the Sema3E–Plexin-D1 signaling axis normalizes angiogenic directionality in both developing retinas and ischemic retinopathy [18]. Neuron-derived Sema3E acts on Plexin-D1-expressing growing blood vessels and activates the small GTPase, RhoJ, in endothelial cells, leading to the suppression of vascular endothelial growth factor (VEGF)-induced filopodia projections. In an oxygen-induced retinopathy model, intravitreal injection of Sema3E selectively suppressed extraretinal vascular outgrowth without affecting the desired regeneration of the retinal vasculature, indicating the therapeutic potential of Sema3E–Plexin-D1 signaling in retinopathy.

In addition to class III semaphorins, the Sema4D–Plexin-B1 signaling axis contributes to the development of diabetic retinopathy [19]. DR patients exhibit increased levels of soluble Sema4D in the aqueous fluid. In mice, streptozotocin-induced diabetes or oxygen-induced retinopathy induces Sema4D expression in the retina. *Sema4d*^*−/−*^ mice show attenuated pathological retinal neovascularization and vascular leakage in a streptozotocin-induced diabetes or oxygen-induced retinopathy model, indicating the pathological roles of Sema4D signaling in retinopathy. Sema4D–Plexin-B1 signaling impairs endothelial cell function through mDIA1, which functions as an adaptor protein of Src kinase. In addition, Sema4D–Plexin-B1 signaling induces pericyte migration and N-cadherin internalization, which exacerbates vascular permeability. Moreover, anti-Sema4D and anti-VEGF antibodies have synergistic effects in inhibiting retinal neovascularization and vascular leakage, indicating the promising potential of Sema4D as a therapeutic target for retinopathy.

Diabetic nephropathy (DN) is the leading cause of hemodialysis worldwide. Hyperglycemia, the primary pathogenic factor responsible for the development of DN, causes hypertension, altered tubuloglomerular feedback, renal hypoxia, and podocyte injury, leading to progressive glomerular sclerosis and impaired glomerular filtration [20]. Sema3A–Plexin-A1 signaling is pathogenic in DN [21]. The expression of Sema3A is upregulated in the podocytes of patients with advanced DN. In streptozotocin-induced diabetic mice, podocyte-specific overexpression of Sema3A disrupts the glomerular filtration barrier and causes massive proteinuria and renal failure, leading to advanced DN. Sema3A induces laminin and collagen IV accumulation in glomerular nodules and causes diffuse podocyte foot process effacement and F-actin collapse via nephrin, αvβ3 integrin, and MICAL1 interactions with Plexin-A1.

In contrast to the pathogenic roles of Sema3A in DN, Sema3G protects podocytes from inflammatory kidney diseases and DN [22]. Although Sema3G is expressed in glomerular podocytes, *Sema3g*^*−/−*^ mice exhibit only partially aberrant podocyte foot process structures without significant glomerular defects. However, *Sema3g*^*−/−*^ mice exhibit increased albuminuria in a lipopolysaccharide (LPS)-induced acute inflammation model or a streptozotocin-induced diabetes model. Moreover, the administration of LPS in mice with podocyte-specific deletion of Sema3G results in enhanced expression of inflammatory cytokines, such as chemokine ligand 2 and interleukin 6. Overall, podocyte-derived Sema3G is protective against kidney inflammation.

Diabetes perturbs bone remodeling and induces osteoporosis [23]. Semaphorin signaling is deeply involved in the maintenance of bone homeostasis [24], indicating a link between semaphorin signaling and skeletal disorders in diabetes. Sema3A plays a pivotal role in osteoprotection by suppressing osteoclastogenesis and promoting osteoblast differentiation and osteocyte survival [25, 26]. Moreover, autocrine Sema3A–Plexin-A4 signaling in neurons maintains bone mass by promoting the sensory innervation of the bone [27]. Sema3A signaling is also involved in the development of diabetic osteoporosis [28]. Diabetes impairs bone formation and strength in rat femurs by suppressing the expression of Sema3A, β-catenin, and IGF-1. The Sema3A–Nrp-1 signaling axis promotes osteoblastogenesis and inhibits adipocyte differentiation through the canonical Wnt/β-catenin signaling pathway [25]. Furthermore, the expression of Sema3A is suppressed in bone mesenchymal stem cells (BMSCs) derived from diabetic rats [29]. Although BMSCs generally possess high osteogenic capacity, diabetic BMSCs exhibit impaired osteogenesis. Sema3A treatment enhances the expression of osteogenesis-related genes, such as those encoding type I collagen, alkaline phosphatase, Runt-related transcription factor 2, bone morphogenetic protein, and osteocalcin, leading to the rescue of osteogenic capacity in diabetic BMSCs. Collectively, the findings of these studies suggest that the Sema3A signaling axis may be a promising therapeutic target for diabetic osteoporosis.

Delayed wound healing is a major complication of diabetes. Delayed and impaired healing causes diabetic foot ulcers, which can result in lower limb amputation [30]. Sema4D exerts protective effects against diabetic wound healing [31]. In leptin receptor-deficient mice, Sema4D suppresses tissue inflammation and promotes angiogenesis, leading to accelerated wound healing. In contrast, Sema6A impairs endothelial sprouting and inhibits diabetic wound healing [32]. Patients with diabetes exhibit decreased expression of miR-27b, which directly targets *Sema6a* mRNA. miR-27b augments bone marrow-derived angiogenic cell function in diabetes by suppressing Sema6A expression, leading to accelerated wound healing. These studies highlight the close association between semaphorin signaling and diabetic wound healing. Due to the suppression of Sema3C–Nrp-2 signaling, diabetes induces corneal dysfunction, such as delayed epithelial wound healing and nerve regeneration [33]. Although wounding upregulates the expression of Sema3C and its receptor Nrp-2 in normal corneal epithelial cells, this upregulation is markedly suppressed in diabetic corneas. Blockade of Sema3C–Nrp-2 signaling impairs epithelial wound healing and corneal nerve regeneration in diabetic corneas, while subconjunctival injection of Sema3C augments wound healing and corneal nerve regeneration in diabetic cornea. Therefore, the Sema3C–Nrp-2 signaling axis plays essential roles in corneal epithelial wound healing and sensory nerve regeneration.

### Role of semaphorins in atherosclerosis

**Fig. 3:**
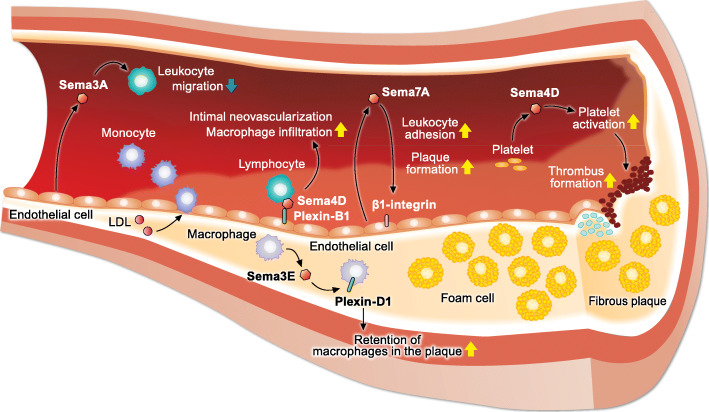
Role of semaphorin signaling in the pathogenesis of atherosclerosis. Sema4D promotes the development of atherosclerosis by promoting thrombus formation, intimal neovascularization, and recruitment of macrophages into atherosclerotic plaques. In addition, Sema7A aggravates plaque formation in atherosclerosis by promoting leukocyte adhesion and vascular inflammation. In contrast, Sema3A inhibits leukocyte rolling, adhesion, and transmigration into atherosclerotic lesions, resulting in the suppression of inflammatory responses in atherosclerosis

Disrupted lipid metabolism causes atherosclerosis, a chronic systemic inflammatory disease characterized by the accumulation of lipids and inflammatory immune cells in the intima of blood vessels. Recent studies have revealed the essential role of semaphorin signaling in the development of atherosclerosis [5] (Fig. 3).

The association between semaphorin signaling and atherosclerosis was first identified in 2009 [34]. Given that Sema4D promotes thrombus formation and growth via platelet activation [35], Zhu et al. [34] investigated the role of Sema4D-mediated platelet activation in the development of atherosclerosis. They revealed that deletion of *Sema4d* reduces dyslipidemia-induced platelet hyperactivity and ameliorates atherosclerosis in low-density lipoprotein receptor (LDLR)-deficient mice fed with a high-fat diet. Our previous study also showed that loss of *Sema4d* in apolipoprotein-E (ApoE)-deficient mice retards the progression of atherosclerosis [36]. Sema4D, which is mainly expressed in infiltrating lymphoid cells in atherosclerotic lesions, promotes intimal neovascularization and macrophage infiltration in atherosclerotic plaques.

Sema3A exerts atheroprotective effects by inhibiting leukocyte rolling, adhesion, and transmigration into the subendothelial wall during atherogenesis [37]. In LDLR-deficient mice fed a Western diet, endothelial cells in the atheroprotective outer curvature highly express Sema3A, while endothelial cells in the atheroprone inner do not express Sema3A. Oscillatory flow, which is characteristic of the atheroprone regions of the vasculature, suppresses Sema3A expression in human coronary artery endothelial cells in vitro. Moreover, Sema3A-blocking peptide increased leukocyte adhesion to the endothelium in vivo. Thus, Sema3A is a gatekeeper for leukocyte invasion into atherosclerotic regions.

The Sema3E–Plexin-D1 signaling axis promotes the development of atherosclerosis via the retention of inflammatory macrophages in the plaque [38]. In ApoE-deficient mice fed a Western diet, Sema3E and its receptor Plexin-D1 are expressed in macrophages of advanced atherosclerotic lesions. During atherosclerosis regression, the expression of Sema3E in plaque macrophages was markedly suppressed, in conjunction with the reduced and enhanced expression of *Nos2* and *Arg1*, respectively. *Nos2* and *Arg1* are marker genes of inflammatory and anti-inflammatory macrophages, respectively. Sema3E binding to Plexin-D1 inhibits the directional migration of macrophages by disrupting the Rho GTPase signaling cascade, actin cytoskeleton reorganization, and polarization. In contrast, the Sema3E–Plexin-D1 signaling axis inhibits the proliferation and migration of vascular smooth muscle cells (VSMCs), which are hallmarks of atherosclerosis and restenosis [39]. In a mouse carotid artery ligation model, during neointimal hyperplasia, VSMCs markedly suppress Sema3E expression. Furthermore, the overexpression of Sema3E in the carotid ligation area markedly attenuates neointima formation, indicating the protective effect of Sema3E in neointimal hyperplasia. Sema3E binding to Plexin-D1 inactivates the Rap1-AKT signaling pathways in VSMCs, leading to the suppression of VSMC migration and proliferation.

Recently, Mehta et al. [40] revealed that Plexin-D1 acts as a force detector in endothelial cells, regulating vascular function and the site-specificity of atherosclerosis. Knockdown of Plexin-D1 attenuates the activation induced by shear stress of the key signaling mediators Akt, ERK1/2, and eNOS. Moreover, anti-Sema3E antibody does not affect the flow-induced activation of signaling cascades, indicating that Plexin-D1-dependent mechanotransduction is independent of its ligand Sema3E. In endothelial cells, Plexin-D1 is a direct force sensor that forms a mechanocomplex with Nrp-1 and VEGFR2, which is necessary and adequate for conferring mechanosensitivity upstream of the junctional complex and integrins. When crossed with hypercholesterolemic *Apoe*^*−/−*^ mice, endothelial-specific Plexin-D1 knockout (*Plxnd1*^*iECKO*^) mice fed a high-fat diet exhibit a significant decrease in the plaque burden of both the whole aorta and the aortic arch. In contrast, *Plxnd1*^*iECKO*^*Apoe*^*−/−*^ mice show an increase in plaque burden in the descending aorta. Thus, the endothelial Plexin-D1–Nrp-1–VEGFR2 complex directly senses shear stress and regulates the site-specificity of atherosclerosis.

In 2018, Hu et al. [41] revealed that the Sema7A–β1-integrin signaling axis promotes the development of atherosclerosis via impaired endothelial function and enhanced vascular inflammation. In *Apoe*^*−/−*^ mice fed a high-fat diet, *Sema7a* deletion attenuates atherosclerotic plaque formation mainly in the aortic arch, an atheroprone region exposed to disturbed blood flow. Disturbed blood flow upregulates vascular endothelial Sema7A expression in the atheroprone lesser curvature. Sema7A enhances the endothelial expression of intercellular adhesion molecule 1 and vascular cell adhesion molecule 1 via β1-integrin, resulting in the promotion of leukocyte adhesion and plaque formation. Another study by Hu et al. [42] has revealed that the serum levels of Sema7A are positively associated with the risk of acute atherothrombotic stroke. Moreover, the Sema7A–β1-integrin signaling axis promotes VEGFA/VEGFR2-mediated angiogenesis and intraplaque neovascularization [43]. Therefore, Sema7A plays an essential role in the development of atherosclerosis via multiple pathways.

## Conclusions

Semaphorin signaling has emerged as a pivotal factor regulating the pathogenesis of metabolic diseases by modulating immune responses, endothelial functions, and neural responses. Although recent advances have elucidated the importance of semaphorin signaling in metabolic disorders, several challenges remain in therapeutically targeting semaphorin signaling. First, semaphorin–plexin interactions are context-dependent and highly redundant, which makes it difficult to selectively target specific ligand-receptor interactions in semaphorin signaling. Second, most of the studies described herein utilized mice with whole body deletion of semaphorin molecules. Given that various types of cells express semaphorins and that their expression is tightly regulated during tissue development, this approach does not clarify the specificity of semaphorin signaling in the context of disease development. In summary, the translation of recent experimental insights into successful clinical interventions requires deciphering the spatiotemporal dynamics of semaphorin signaling, and developing reagents that selectively activate or inhibit semaphorin signaling.

## Data Availability

Not applicable.

## Abbreviations

Nrp: Neuropilin
PI3K: Phosphatidylinositol-3 kinase
GSK3: Glycogen synthase kinase 3
AMPK: AMP-activated protein kinase
SREBP-1c: Sterol regulatory element binding protein-1c
POMC: Pro-opiomelanocortin
ARH: Arcuate nucleus of the hypothalamus
PVH: Paraventricular nucleus of the hypothalamus
BAT: Brown adipose tissue
DR: Diabetic retinopathy
VEGF: Vascular endothelial growth factor
DN: Diabetic nephropathy
LPS: Lipopolysaccharide
BMSC: Bone mesenchymal stem cell
LDLR: Low-density lipoprotein receptor
ApoE: Apolipoprotein-E
VSMC: Vascular smooth muscle cell
